# Supplementary material for: MetaRibo-Seq measures translation in microbiomes
Source: Nat Commun. 2020 Jun 29;11:3268. doi: 10.1038/s41467-020-17081-z (PMC7324362; doi:10.1038/s41467-020-17081-z)
Supplement: Supplementary file 10 — Supplementary Data 7 [file 41467_2020_17081_MOESM10_ESM.zip › File2/Confidence_High_Taxonomy/199766_out.krona.html]

Javascript must be enabled to view this page.

members
magnitude
magnitudeUnassigned
count
unassigned
taxon
rank

199766\_out

185

superkingdom
185
2

976
4
phylum

class
4
200643

order
4
171549

family
4
815

genus
816
4

species

SRS015264\_contig\_number\_23062SRS046717\_contig\_number\_contig-100\_4495.90340SRS049900\_contig\_number\_18238SRS146812\_contig\_number\_30016
4
384639

181
1239
phylum

181
186801
class

181

SRS016897\_contig\_number\_contig-100\_10153.10154SRS024663\_contig\_number\_11384SRS058770\_contig\_number\_18327SRS146764\_contig\_number\_contig-100\_6101.212466SRS148817\_contig\_number\_contig-100\_2990.2991SRS149784\_contig\_number\_contig-100\_4711.90847SRS893342\_contig\_number\_5441
order
186802
7

47
186803
family

SRS011084\_contig\_number\_contig-100\_369.214109SRS013476\_contig\_number\_contig-100\_2607.218815SRS013965\_contig\_number\_contig-100\_3650.226567SRS014235\_contig\_number\_contig-100\_5041.261247SRS015065\_contig\_number\_12803SRS019161\_contig\_number\_26051SRS020233\_contig\_number\_contig-100\_1190.343537SRS022609\_contig\_number\_27924SRS024132\_contig\_number\_52492SRS045004\_contig\_number\_8101SRS049896\_contig\_number\_29757SRS050752\_contig\_number\_contig-100\_3616.176994SRS050925\_contig\_number\_14895SRS052697\_contig\_number\_contig-100\_636.259466SRS053356\_contig\_number\_contig-100\_2859.277825SRS062427\_contig\_number\_contig-100\_2060.79459SRS064557\_contig\_number\_contig-100\_2249.128608SRS075821\_contig\_number\_15943SRS076929\_contig\_number\_25930SRS077024\_contig\_number\_1557SRS077641\_contig\_number\_2115SRS077730\_contig\_number\_5656SRS077849\_contig\_number\_25721SRS097920\_contig\_number\_contig-100\_4090.196870SRS098571\_contig\_number\_61417SRS098655\_contig\_number\_6328SRS1041031\_contig\_number\_contig-100\_2044.162558SRS1041033\_contig\_number\_contig-100\_1729.146815SRS1041118\_contig\_number\_contig-100\_9152.9153SRS1041137\_contig\_number\_contig-100\_7372.147149SRS1041145\_contig\_number\_20515SRS1055034\_contig\_number\_contig-100\_3123.81186SRS1055038\_contig\_number\_2615SRS1055069\_contig\_number\_contig-100\_230.100178SRS143372\_contig\_number\_12636SRS143466\_contig\_number\_27799SRS144297\_contig\_number\_9295SRS144362\_contig\_number\_9116SRS144362\_contig\_number\_24041SRS144603\_contig\_number\_contig-100\_1875.105509SRS147139\_contig\_number\_45336SRS148159\_contig\_number\_contig-100\_3693.314782SRS148784\_contig\_number\_contig-100\_1404.169492SRS893187\_contig\_number\_17384SRS893358\_contig\_number\_7489SRS893369\_contig\_number\_contig-100\_1934.73766SRS893383\_contig\_number\_contig-100\_5316.209442
173

1898203
8

SRS019808\_contig\_number\_18116SRS048060\_contig\_number\_1632SRS051882\_contig\_number\_13246SRS063985\_contig\_number\_23193SRS1041038\_contig\_number\_16324SRS1041133\_contig\_number\_12200SRS893285\_contig\_number\_1876SRS971276\_contig\_number\_23964
species

genus
572511
1

1
40520

SRS017307\_contig\_number\_20463
species

genus
3
830

species
1262761
3

SRS013687\_contig\_number\_15748SRS054905\_contig\_number\_23382SRS148319\_contig\_number\_contig-100\_269.101084

species
2109691
2

SRS062701\_contig\_number\_7086SRS1041142\_contig\_number\_8493

species
39491
39

SRS011239\_contig\_number\_9839SRS012849\_contig\_number\_16993SRS013940\_contig\_number\_18857SRS014613\_contig\_number\_3325SRS014683\_contig\_number\_14951SRS014923\_contig\_number\_13274SRS015217\_contig\_number\_19049SRS015794\_contig\_number\_contig-100\_152.94235SRS017103\_contig\_number\_42633SRS019030\_contig\_number\_16332SRS021948\_contig\_number\_35986SRS022137\_contig\_number\_18629SRS024435\_contig\_number\_25692SRS049446\_contig\_number\_contig-100\_504.93542SRS053214\_contig\_number\_22703SRS053398\_contig\_number\_12483SRS055017\_contig\_number\_33459SRS056519\_contig\_number\_18838SRS058070\_contig\_number\_7034SRS063985\_contig\_number\_31789SRS074586\_contig\_number\_contig-100\_87.58500SRS075341\_contig\_number\_25988SRS077231\_contig\_number\_26627SRS077552\_contig\_number\_29213SRS098717\_contig\_number\_contig-100\_86.150239SRS1041036\_contig\_number\_1635SRS1041144\_contig\_number\_7565SRS1055056\_contig\_number\_9879SRS142542\_contig\_number\_15872SRS142890\_contig\_number\_10588SRS143417\_contig\_number\_33999SRS143991\_contig\_number\_30847SRS144714\_contig\_number\_28662SRS148253\_contig\_number\_2850SRS149244\_contig\_number\_6079SRS149325\_contig\_number\_14868SRS893231\_contig\_number\_3129SRS893279\_contig\_number\_contig-100\_115.102170SRS893373\_contig\_number\_contig-100\_46.156417

genus
71
841

species

SRS011134\_contig\_number\_29124SRS013951\_contig\_number\_3037SRS015578\_contig\_number\_14676SRS016989\_contig\_number\_17121SRS017191\_contig\_number\_860SRS017916\_contig\_number\_17426SRS019068\_contig\_number\_61155SRS019496\_contig\_number\_18075SRS020869\_contig\_number\_1270SRS024331\_contig\_number\_15091SRS053335\_contig\_number\_872SRS055966\_contig\_number\_contig-100\_6430.6431SRS078419\_contig\_number\_contig-100\_2769.223210SRS097889\_contig\_number\_27733SRS098571\_contig\_number\_contig-100\_3394.44126SRS103987\_contig\_number\_30628SRS104693\_contig\_number\_10866SRS1055076\_contig\_number\_contig-100\_461.99299SRS142781\_contig\_number\_contig-100\_103.90676SRS144135\_contig\_number\_32554SRS146812\_contig\_number\_967SRS147271\_contig\_number\_19243SRS147614\_contig\_number\_8125SRS148424\_contig\_number\_15749SRS149075\_contig\_number\_18692SRS893341\_contig\_number\_14857SRS893378\_contig\_number\_438
27
1897016


SRS016095\_contig\_number\_13736SRS142980\_contig\_number\_7324
1262940
2
species

species
360807
8

SRS013158\_contig\_number\_4328SRS014459\_contig\_number\_8110SRS016132\_contig\_number\_contig-100\_94.95SRS019445\_contig\_number\_609SRS024492\_contig\_number\_12100SRS077502\_contig\_number\_contig-100\_27851.27851SRS144506\_contig\_number\_39142SRS893230\_contig\_number\_contig-100\_1462.72809

species

SRS015190\_contig\_number\_contig-100\_184.101117SRS015960\_contig\_number\_20357SRS016018\_contig\_number\_contig-100\_34.103048SRS017821\_contig\_number\_contig-100\_1163.196063SRS018817\_contig\_number\_3846SRS019397\_contig\_number\_3584SRS024388\_contig\_number\_1927SRS024549\_contig\_number\_5518SRS042628\_contig\_number\_20841SRS043001\_contig\_number\_11664SRS043411\_contig\_number\_contig-100\_249.109161SRS056259\_contig\_number\_25261SRS063518\_contig\_number\_30292SRS075716\_contig\_number\_3902SRS076756\_contig\_number\_21495SRS098061\_contig\_number\_13851SRS098073\_contig\_number\_contig-100\_1824.115646SRS098881\_contig\_number\_1534SRS101433\_contig\_number\_contig-100\_1538.111163SRS104084\_contig\_number\_11373SRS1041130\_contig\_number\_1653SRS143070\_contig\_number\_11215SRS149181\_contig\_number\_contig-100\_51.185330SRS893172\_contig\_number\_3094
24
166486

species
1
2293144

SRS893270\_contig\_number\_1210


SRS018836\_contig\_number\_15983
1
2292367
species

4
301301

SRS014923\_contig\_number\_28240SRS015431\_contig\_number\_41775SRS015579\_contig\_number\_19985SRS063127\_contig\_number\_388
species

species
2
2049040

SRS023526\_contig\_number\_21037SRS146812\_contig\_number\_contig-100\_3076.46417

2
1897017

SRS050941\_contig\_number\_15315SRS143780\_contig\_number\_12662
species

2
189330
genus

39486
2

SRS052027\_contig\_number\_10979SRS1055022\_contig\_number\_18926
species

family
1
186806

1
1730
genus

species
1
1263078

SRS148721\_contig\_number\_41826
